# The role of post‐translational modifications in the dynamics of cytoplasmic biomolecular condensates in plants

**DOI:** 10.1111/nph.70593

**Published:** 2025-09-25

**Authors:** Margaux Legoux, Jean‐Philippe Reichheld, Rémy Merret

**Affiliations:** ^1^ CNRS‐LGDP UMR 5096 58 Avenue Paul Alduy 66860 Perpignan France; ^2^ Université de Perpignan Via Domitia, LGDP‐UMR5096 58 Avenue Paul Alduy 66860 Perpignan France; ^3^ IBMP UPR 2357 23 rue Général Zimmer 67000 Strasbourg France

**Keywords:** cytoplasmic biomolecular condensates, granules, liquid–liquid phase separation, post‐translational modifications, stress

## Abstract

Cytoplasmic biomolecular condensates are membrane‐less structures important for cell homeostasis, especially during stress conditions. These aggregates concentrate RNA, protein, and metabolites during a wide variety of stresses. The formation of cytoplasmic biomolecular condensates was studied extensively in plants, revealing many key actors involved in their nucleation. More recently, post‐translational modifications (PTMs) of some of these components appear as a novel layer in cytoplasmic biomolecular condensate formation. Here, we describe the importance of the PTMs in cytoplasmic biomolecular condensate dynamics in plants. We highlight the major contribution of phosphorylation and ubiquitination in these processes and discuss the involvement of other recently discovered PTMs.

## Introduction

Environmental stresses, such as temperature fluctuations, drought, salt, and oxidative stress, trigger a wide range of physiological and molecular changes in all living organisms. Like other organisms, plants have evolved complex and highly regulated responses to cope with these environmental challenges. One critical aspect of the cellular stress response involves the protection and regulation of essential macromolecules, including DNA, RNA, and proteins. Among these, the regulation of messenger RNA (mRNA) metabolism has emerged as a particularly dynamic and responsive layer of gene expression control (Xiang & Dong, 2025). This regulation allows plants to swiftly adapt their proteome in response to changing environmental conditions by modulating mRNA translation, stability, and localization.

In eukaryotic cells, untranslated or translationally repressed mRNAs often aggregate with RNA‐binding proteins and other associated factors to form cytoplasmic structures known as messenger ribonucleoprotein (mRNP) granules. These dynamic assemblies, also referred to as biomolecular condensates, represent membrane‐less organelles that compartmentalize biochemical reactions and modulate gene expression in response to cellular needs (Banani *et al*., 2017). The term ‘biomolecular condensates’ broadly encompasses various condensate types that form via a biophysical process called liquid–liquid phase separation (LLPS), enabling the selective concentration of specific molecules within discrete cellular domains. Recent research has highlighted the central role of biomolecular condensates in plant stress responses (Solis‐Miranda *et al*., 2023). In particular, cytoplasmic biomolecular condensates such as processing bodies (PBs), stress granules (SGs), and siRNA‐bodies have been implicated in diverse aspects of plant development and stress adaptation (Field *et al*., 2023). These structures serve not only as sites of mRNA storage, degradation, or translational control but also as dynamic regulators that help to buffer the cellular effects of stress.

A major breakthrough in our understanding of biomolecular condensate biology has been the discovery that their assembly and disassembly are tightly regulated by post‐translational modifications (PTMs) of key protein components (Kearly *et al*., 2024). PTMs such as phosphorylation, ubiquitination, acetylation, and SUMOylation can modulate the physicochemical properties of proteins, influencing their solubility, interaction networks, subcellular localization, and activity. These reversible modifications provide a rapid and reversible mechanism for cells to remodel condensate composition and dynamics in response to external cues. In the context of environmental stress, PTMs represent a powerful means by which plants can fine‐tune condensate behavior and, by extension, mRNA metabolism and metabolic processes. These modifications can alter the biochemical properties of proteins, their localization, activity, or interaction with other macromolecules (Vu *et al*., 2018).

In this review, we aim to synthesize recent advances in our understanding of how PTMs regulate the formation, composition, and function of cytoplasmic biomolecular condensates in plants. We will explore the molecular mechanisms through which PTMs influence condensate dynamics, highlight emerging examples of stress‐responsive PTM‐mediated regulation, and discuss the broader implications of this regulation for plant stress tolerance and resilience.

## Techniques used to characterize cytoplasmic biomolecular condensate composition

In recent years, significant progress has been made in developing robust techniques to characterize the molecular composition of cytoplasmic biomolecular condensates. In plants, initial insights were gained through mass spectrometry analyses of interactors associated with core granule components. These studies utilized transgenic lines combined with immunoprecipitation approaches (Gutierrez‐Beltran *et al*., 2015, 2021; Lokdarshi *et al*., 2016). As an example, Tudor Staphylococcal Nuclease 2 (TSN2) was used as an SG marker and allows the identification of 148 stress‐dependent interactors potentially interacting in SG (Gutierrez‐Beltran *et al*., 2021). To improve the specificity and enrichment of granule‐associated proteins, some studies integrated immunoprecipitation with granule purification techniques based on differential centrifugation (Kosmacz *et al*., 2019; Li *et al*., 2024; Wang *et al*., 2024; Xie *et al*., 2024; Q. Chen *et al*., 2024). More recently, proximity labeling methods based on TurboID have been employed as a powerful alternative for capturing the dynamic and spatially restricted proteomes of cytoplasmic condensates (Liu *et al*., 2023, 2024). Collectively, these approaches extend the large diversity of proteins identified in cytoplasmic condensates (reviewed in (Solis‐Miranda *et al*., 2023)). Intriguingly, tens of proteins not directly linked to RNA biology have also been consistently identified, suggesting broader cellular functions for these condensates.

**Fig. 1:**
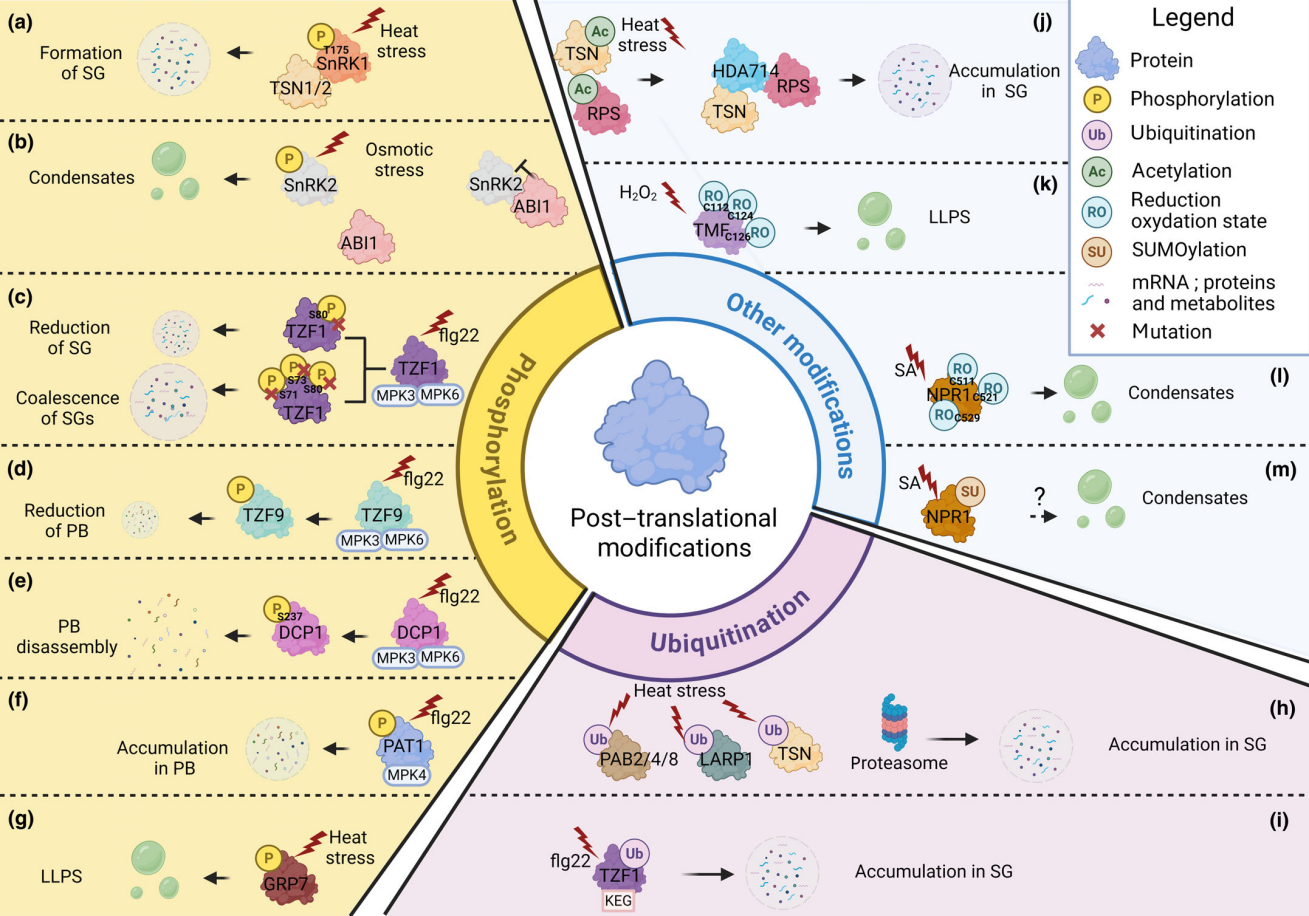
Impact of post‐translational modifications on cytoplasmic biomolecular condensate dynamics. The figure shows the importance of phosphorylation (a–g), ubiquitination (h, i), and other PTMs (j–m) in the dynamics of cytoplasmic biomolecular condensate formation under different biotic and abiotic stresses and for different target proteins. Phosphorylation can lead to the formation or coalescence of stress granules (a). Mutations in specific phosphosites can lead to opposite effects such as stress granule reduction or stress granule coalescence (c). Phosphorylation can also lead to condensate formation by liquid–liquid phase separation (b, g). Phosphorylation can induce the accumulation of target proteins in processing bodies (f) or, conversely, lead to the reduction or disassembly of processing bodies (d, e). Ubiquitination appears to play a positive role in the formation of stress granules since, under stress conditions, many target proteins are ubiquitinated, leading to their accumulation in stress granules (h, i). The proteasome is also present in stress granules (h). Other modifications, such as deacetylation of certain proteins, lead to their accumulation in stress granules (j); modification of the redox state of specific cysteines and potentially SUMOylation are also involved in condensate formation via liquid–liquid phase separation (k–m). flg22, Flagellin peptide flg22; H_2_O_2_, hydrogen peroxide; KEG, KEEP‐ON‐GOING; LLPS, liquid–liquid phase separation; MPK3/6, Mitogen‐activated protein kinases 3 and 6; PB, processing bodies; SA, Salicylic acid; SG, stress granules. This figure was created in BioRender. Legoux, M. (2025) https://BioRender.com/4e9qtk4.

Beyond proteins, diverse small molecules have been found within cytoplasmic condensates. These include phospholipids, polar metabolites such as amino acids and nucleotides (Kosmacz *et al*., 2018, 2019; Chodasiewicz *et al*., 2022; Song *et al*., 2024). While not localized in condensates, some metabolites were also proposed to participate in condensate formation and dynamics. 2′,3′‐cAMP has been proposed to play roles in SG formation and PB dynamics (Kosmacz *et al*., 2018, 2019; Chodasiewicz *et al*., 2022). Additionally, energy‐related molecules such as ATP and NAD^+^ have been shown to influence protein condensation behavior, possibly acting as modulators of phase separation (Song *et al*., 2024). More precisely, the formation of TIR condensates (N‐terminal Toll/interleukin‐1 receptor) is mediated by ATP and NAD^+^. The direct binding of ATP and NAD^+^ to TIR domain protein is sufficient to induce condensation *in vitro* (Song *et al*., 2024). Enzymatic components are also prevalent in these condensates. For example, enzymes involved in protein dephosphorylation/phosphorylation (such as MAP kinases), ethylene pathway (such as ACC oxidase), glutathione pathway (such as glutathione peroxidase), or glycolysis (such as enolase) were identified in SGs or PBs (Solis‐Miranda *et al*., 2023), highlighting the potential importance of catalytic processes in condensate assembly and function. In the following sections, we will explore how PTMs contribute to the regulation of cytoplasmic biomolecular condensate dynamics (Fig. 1; Table 1). Our survey reveals that PTMs of both RNA‐binding and non‐RNA‐binding proteins contribute to condensate dynamics in plants.

**Table 1:** Summary of the different post‐translational modifications implicated in condensate dynamics in plants: (a) Phosphorylation; (b) ubiquitination; (c) acetylation; (d) redox regulation.

Protein	Fonction	Impacts	Condensates	Stress	References
(a) Phosphorylation					
SnRK1	Kinase	TSN interacts with SnRK1 and enhances its kinase activity, the phosphorylation of SnRK1 mediates its assembly in SG.	Stress Granules	Heat	Gutierrez‐Beltran *et al*. (2021)
SnRK2	Kinase	SnRK2 dissociates from the negative regulator ABI1 by the formation of condensates wich conduce to it's activation. This activation can be modulated by a protein kinase which localizes into PB during stress.	Crowding condensates	Osmotic	Yuan & Zhao (2025)
TZF1	RBP	Phosphorylation of TZF1 via MPK3 and 6 regulates the dynamics of SG formation.	Stress Granules	Biotic	He *et al*. (2024)
TZF9	RBP	Phosphorylation of TZF9 via MKP3 and MPK6 leads to reduction of PB and RNA‐binding properties.	Processing bodies	Biotic	Tabassum *et al*. (2020)
DCP1	RBP	DCP1 phophorylation at serine 237 via MPK3 and PK6 is required for PB disassembly.	Processing bodies	Biotic	Yu *et al*. (2019)
PAT1	RBP	Phosphorylation of PAT1 via MPK4 induces its localization into PB.	Processing bodies	Biotic	Roux *et al*. (2015)
GRP7	RBP	GPR7 phosphorylation by FERONIA leads to LLPS separation and is link to heat stress tolerance. GRP7 recruits mRNAs and translation machinery which is essential for SG formation.	LLPS	Heat	Xu *et al*. (2024)
(b) Ubiquitination					
Proteasome constituents	Proteasome	Ubiquitination is needed for SG disassembly.	Stress granules	Heat	Xie *et al*. (2024)
HSP101	Chaperone	HSP101 and the proteasome are involved in the solubilization and degradation of ubiquitylated proteins during recovery after stress, needed for plant survival to proteotoxic stress.	Stress granules	Heat	McLoughlin *et al*. (2019)
AP‐3β	Trafficking	The 19S regulatory particle is recruited to facilitate SG disassembly.	Stress granules	Heat	Pang *et al*. (2025)
Autophagy proteins	Autophagy	After heat stress, the disassembly of SG allows autophagy proteins to relocate in the cytoplasm, enabling fast elimination of ubiquitinated aggregates. In SG‐deficient mutant, this activation is delayed leading to inefficient removal of ubiquitinated proteins.	Stress granules	Heat	Li *et al*. (2024)
TZF1	RBP	TZF1 is ubiquitinated inducing its accumulation to SG. Mutation of TZF1 ubiquitination sites affect SG assembly.	Stress granules	Biotic	He *et al*. (2024)
(c) Acetylation					
HDA714	Histone deacetylase	HDA714 deacetylates some SG proteins influencing their aggregation.	Stress granules	Heat	Q. Chen *et al*. (2024), Z. Chen *et al*. (2024)
(d) Redox regulation					
TMF	Transcription factor	The production of hydrogen peroxide leads to liquid–liquid phase separation of TMF in tomato meristem.	Nuclear LLPS	Oxidative	Huang *et al*. (2021)
NPR1	Signaling	NPR1 cysteines in or nearby IDR regions leads to the formation of condensates.	Condensates	Biotic	Zavaliev *et al*. (2020)

ABI1, ABA‐insensitive 1; AP‐3β, β subunit of adaptor protein −3 complex; DCP1, decapping protein 1; GRP7, glycine‐rich protein 7; HSP101, heat shock protein 101; HDA714, histone deacetylase 714; LLPS, liquid–liquid phase separation; MPK3, mitogen‐activated protein kinase 3; MPK6, mitogen‐activated protein kinase 6; NPR1, non‐expresser of pathogenesis related PR1; PAT1, protein associated with topoisomerase II; PB, processing‐bodies; RBP, RNA‐binding protein; SnRK1, Sucrose nonfermenting 1‐related protein kinase 1; SnRK2, Sucrose nonfermenting 1–related protein kinase 2; SG, Stress Granule; TMF, Terminating Flower; TZF1, tandem CCCH zinc finger 1; TZF9, tandem CCCH zinc finger 9.

## Phosphorylation‐mediated dynamics of cytoplasmic biomolecular condensates

Recent studies have identified several enzymes involved in phosphorylation and dephosphorylation within cytoplasmic biomolecular condensates, such as SG and PB (Lokdarshi *et al*., 2016; Kosmacz *et al*., 2019; Gutierrez‐Beltran *et al*., 2021; Solis‐Miranda *et al*., 2023; Yuan & Zhao, 2025) (Fig. 1a,b; Table 1a). A key example involves SnF1‐Related protein kinases 1 and 2 (SnRK1 and SnRK2), central components of stress signaling pathways in plants. Both kinases have been detected in distinct condensates – SnRK1 localizing predominantly to SGs and SnRK2 to PBs in *Arabidopsis thaliana* (Gutierrez‐Beltran *et al*., 2021; Yuan & Zhao, 2025) (Fig. 1a,b). Under heat stress, the TSN1 and 2 physically interact with SnRK1 to facilitate its condensation and activation within SGs (Fig. 1a). Notably, heat‐induced phosphorylation of SnRK1α at threonine 175 is abolished in a *tsn1 tsn2* double mutant, which also shows a marked reduction in SnRK1 accumulation in SGs. This finding underscores the critical role of SG‐mediated kinase localization in plant stress signaling. SnRK2 proteins, on the other hand, were recently proposed as a molecular crowding sensor. Under normal conditions, SnRK2s are associated with ABI1, their negative regulator ABA‐insensitive 1, to prevent activation of a stress response. Upon stress exposure, SnRK2s form condensates that enable their dissociation from ABI1, leading to activation of a stress response and subsequent initiation of stress signaling (Yuan & Zhao, 2025) (Fig. 1b). Moreover, SnRK2s' activity is also modulated by B4 Raf‐like protein kinases, which localize to PBs during stress (Lin *et al*., 2020; Soma *et al*., 2020). This activation by segregation allows the regulation of stress‐responsive genes under severe osmotic stress. In fact, *snrk2* mutants exhibit growth defects under osmotic stresses, more particularly during seed germination (Yuan & Zhao, 2025). These findings mirror observations in mammalian systems, where condensation of kinases and phosphatases is a well‐established stress response mechanism (Su *et al*., 2016; Watanabe *et al*., 2021). This suggests that condensation‐mediated control of kinase/phosphatase activity may be a conserved and universal strategy across kingdoms.

Beyond signaling proteins, phosphorylation has also been implicated in regulating the assembly, dynamics, and size of biomolecular condensates through modifications of RNA‐binding proteins (Fig. 1c–g; Table 1a). For instance, phosphorylation of tandem CCCH zinc Finger proteins by MAP kinases MPK3 and MPK6 modulates their behavior in condensates (Tabassum *et al*., 2020; He *et al*., 2024). In the case of TZF1, specific phosphorylation at serines 71, 73, and 80 is triggered by treatment with the flagellin peptide flg22 of *Pseudomonas syringae*. Mutating these sites leads to aberrant SG fusion and oversized condensates (Fig. 1c), linking phosphorylation to condensate size homeostasis. Similarly, TZF9 undergoes multi‐site phosphorylation by MPK3/6 following flg22 perception, reducing its localization to PB and SG (Tabassum *et al*., 2020) (Fig. 1d). As TZF9 interacts with the poly(A)‐Binding protein 2 (PAB2) localized in SG during stress, it was proposed that its phosphorylation contributes to the translational control of defense mRNAs, while direct evidence is missing (Tabassum *et al*., 2020). This phosphorylation regulatory mechanism appears to extend to RNA decay factors. For example, flg22, known to elicit a specific innate immune response, induces phosphorylation of decapping protein 1 (DCP1) at serine 237, which is required for PB disassembly (Yu *et al*., 2019) (Fig. 1e). DCP1 phosphorylation was proposed to trigger mRNA decay involved in biotic stress responses. Conversely, flg22‐dependent phosphorylation of the mRNA decay factor protein associated with topoisomerase II (PAT1) by the MAP kinase (MPK4) promotes its recruitment to PBs (Roux *et al*., 2015) (Fig. 1f). These latter examples highlight that flg22 induces phosphorylation of multiple substrate proteins to enhance or suppress their association with condensates (Fig. 1c–f). Therefore, phosphorylation might constitute a way to modify the composition of PB under stress conditions.

Heat stress also modulates phase separation through phosphorylation. The Glycine‐Rich RNA‐binding protein GRP7 is phosphorylated in response to elevated temperatures, promoting its condensation in Arabidopsis (Xu *et al*., 2024) (Fig. 1g). Notably, natural variations in GRP7 phosphosites influence its condensation behavior and correlate with temperature adaptation, implying that GRP7 phase separation plays a vital role in thermotolerance. GRP7 phosphorylation appears essential to the formation of SG to recruit mRNA and the translation machinery components to inhibit translation upon heat stress (Xu *et al*., 2024). Furthermore, emerging evidence suggests that light signaling can also impact phosphorylation‐dependent condensation, particularly within the nucleus (Feng *et al*., 2024), expanding the role of PTM to nuclear condensates. In mammals, phosphorylation plays a dual role in condensate formation, particularly in the assembly of SGs, depending on the specific target protein. For instance, Ras GTPase‐activating protein‐binding protein 1 (G3BP1), a key factor in SG nucleation, can be phosphorylated at serine 149 by Casein Kinase 2, leading to a reduction in SG formation (Reineke *et al*., 2017). Conversely, phosphorylation can also promote SG assembly; for example, phosphorylation of eukaryotic Initiation Factor 2α (eIF2α) by specific stress‐activated kinases facilitates SG formation (Ryan & Rubinsztein, 2024). Similarly, in plants, phosphorylation acts as a regulatory mechanism that can either enhance or inhibit SG formation, depending on the nature of the stress, the identity of the protein, and the phosphorylation site involved. Taken together, these findings highlight the central role of phosphorylation in modulating the behavior of biomolecular condensates during environmental stress. From signal transduction to RNA metabolism, phosphorylation serves as a versatile regulatory mechanism governing protein localization, activity, and condensate organization in plant cells.

## Ubiquitination‐mediated dynamics of cytoplasmic biomolecular condensates

In addition to phosphorylation, ubiquitination also appears important for cytoplasmic biomolecular condensate dynamics (Fig. 1h,i; Table 1b). The first evidence of the importance of ubiquitination was reported during heat stress in Arabidopsis (McLoughlin *et al*., 2019). Analysis of insoluble proteins during heat stress reveals that those proteins are extensively ubiquitylated, especially many RNA‐binding proteins known as SG components such as the poly(A) binding proteins PAB2/4/8, the TSN1/2, or the La‐related protein 1 LARP1 (Fig. 1h). This large ubiquitination was recently confirmed (Xie *et al*., 2024) and is consistent with the presence of proteasome constituents in SG (McLoughlin *et al*., 2019; Xie *et al*., 2024). More particularly, heat stress triggers extensive ubiquitylation of SG components. Proteolytic activity in SG triggers their disassembly during the recovery phase, essential for translation recovery and heat tolerance (McLoughlin *et al*., 2019; Xie *et al*., 2024; Q. Chen *et al*., 2024; Pang *et al*., 2025). In fact, a *pad1* mutant (a subunit of the 20S core particle of the proteasome) exhibits a significantly reduced SG disassembly and displays a heat tolerance phenotype during the recovery phase. How ubiquitylated SG components regulate SG dynamics awaits further exploration (Xie *et al*., 2024). More recently, autophagy activation during heat stress was also proposed to participate in this phenomenon, demonstrating the importance of clearance of ubiquitinated aggregated proteins for stress recovery (Li *et al*., 2024). Notably, in an Oligouridylate‐Binding Protein 1 (UBP1) triple mutant (*ubp1abc*), key components of SG formation, a delay in autophagy activation is observed. This results in a defect in the removal of ubiquitinated proteins produced during heat stress (Li *et al*., 2024).

Some proteins can undergo several PTMs, which have an impact on their granule formation dynamics. For example, TZF1, phosphorylated during flg22 treatment (Fig. 1c), was also reported to be ubiquitinated by an E3 ubiquitin ligase KEEP‐ON‐GOING (KEG) (He *et al*., 2024). TZF1 ubiquitination induces its accumulation to SG (Fig. 1i). Mutations of TZF1 ubiquitination sites drastically affect SG assembly (He *et al*., 2024). In mammals, the importance of ubiquitination and autophagy was also demonstrated. Mutations implicated in several diseases compromise autophagy, resulting in the persistence of condensates (see (Ryan & Rubinsztein, 2024) for a complete review). Altogether, these different results illustrate the importance of ubiquitination and the clearance of ubiquitinated proteins in condensate dynamics and stress response.

## Other PTMs involved in the dynamics of cytoplasmic biomolecular condensates

Lysine acetylation is another PTM mediating cytoplasmic biomolecular condensate metabolism (Fig. 1j; Table 1c). In rice, the cytosolic Histone Deacetylase HDA714 confers plant tolerance to heat stress by controlling the protein lysine acetylation status of several glycolysis enzymes. Upon heat stress, HDA714 accumulates in SG and affects the lysine acetylation status of several SG proteins like TSN and small ribosomal proteins, influencing their own allocation in SG (Z. Chen *et al*., 2024) (Fig. 1j). Although the function of protein lysine acetylation in protein allocation to SG is unclear, previous studies indicated that lysine acetylation may promote protein polyubiquitination and degradation (Du *et al*., 2010; Xu *et al*., 2021). A possible coordination of lysine acetylation and ubiquitination in controlling SG dynamics is still to be investigated. In mammals, the cytosolic histone deacetylase HDAC6 was shown to regulate SG formation by deacetylating the RNA helicase DDX3X and the scaffold protein Ras GTPase‐activating protein binding protein 1 (G3BP1), allowing their localization in SG. It was demonstrated that an N‐terminal Intrinsically Disordered Region (IDR) of DDX3X (IDR1) can undergo LLPS *in vitro*, and its acetylation at multiple lysine residues impairs the formation of liquid droplets. Hence, an enhanced LLPS propensity through the deacetylation of DDX3X‐IDR1 by HDAC6 is necessary for SG maturation (Kwon *et al*., 2007; Saito *et al*., 2019). Redox regulation of cytoplasmic biomolecular condensate metabolism has been shown to regulate flower meristem development in tomato (Fig. 1k; Table 1d). Huang *et al*. (2021) demonstrated that developmentally produced hydrogen peroxide (H_2_O_2_) in the shoot apical meristem (SAM) triggers reversible protein phase separation of TERMINATING FLOWER (TMF), a transcription factor that regulates flowering transition by repressing the pre‐maturation of SAM. Specific cysteine residues (Cys112, 124, and 126) within TMF sense the cellular redox environment to form disulfide bonds that concatenate multiple TMF molecules and elevate the amount of intrinsically disordered regions to trigger phase separation (Fig. 1k). Moreover, redox‐dependent phase separation enables TMF to bind and sequester the promoter of a floral identity gene *ANANTHA* and repress its expression (Huang *et al*., 2021). In mammals, the capacity of TIA1, a core stress granule protein, to localize in SG is redox‐dependent. Reactive oxygen species (ROS) such as H_2_O_2_ oxidize the conserved Cys36 in TIA1 and inhibit SG assembly by impairing its RNA‐binding activity. Hence, suppression of SG formation by oxidative stress underlies neuronal cell death in neurodegenerative diseases (Arimoto‐Matsuzaki *et al*., 2016). Interestingly, the redox‐regulated Cys is conserved in UBP1 proteins, plant homologs of TIA1, suggesting that redox regulation of UBP1 might occur in plants.

Other types of cytosolic condensates have been shown to be under redox regulation. In plants, a salicylic acid (SA)‐induced transition of Non‐expressor of Pathogenesis Related 1 (NPR1) to cytosolic condensates is required for the formation of the NPR1‐Cullin 3 E3 ligase complex to ubiquitinate and promote cell survival under pathogen effector‐triggered immunity. This transition is mediated by conserved redox‐sensitive cysteines (Cys511, 521, and 529) present within NPR1 IDRs. It was proposed that SA triggers a conformational change in NPR1 through modulation of the redox state of these cysteine residues (Fig. 1l). Meanwhile, NPR1 is also prone to SUMOylation in the nucleus, affecting its subsequent ubiquitination and degradation, and also interplaying with condensate formation in the cytoplasm (Fig. 1m) (Zavaliev *et al*., 2020). In mammals, fused in sarcoma (FUS), a DNA/RNA‐binding protein, is associated with neurodegenerative diseases such as amyotrophic lateral sclerosis (ALS) and frontotemporal dementia (FTD). Under neurodegenerative diseases, stress‐induced ROS generation triggers excessive glutathionylation (binding of a glutathione residue) at the Cys447 residue in the DNA‐binding domain of FUS. This modification was proposed to induce conformational changes that favor FUS aggregation by promoting LLPS. Moreover, the glutathione transferase O2 (GstO2) reduced cytoplasmic FUS aggregation by deglutathionylation, and the overexpression of a GstO2 homolog attenuates FUS‐induced neurotoxicity and cytoplasmic FUS accumulation in mouse neuronal cells. Thus, the modulation of FUS glutathionylation was proposed to be a promising therapeutic strategy for FUS‐associated neurodegenerative diseases (Cha *et al*., 2022).

## Additional PTMs involved in the dynamics of cytoplasmic biomolecular condensates identified in other organisms

In other organisms, phosphorylation and ubiquitination have also been shown to play key roles in condensate dynamics (see (Wang *et al*., 2023), for a comprehensive review). In addition, several other PTMs have been described. SUMOylation, a PTM structurally similar to ubiquitination, has been well characterized in mammals and is proposed to influence SG disassembly (Marmor‐Kollet *et al*., 2020). Impaired SUMOylation delays SG disassembly, a phenomenon observed in neurodegenerative diseases (Marmor‐Kollet *et al*., 2020). NEDDylation, the addition of an NEDD8 polypeptide, has also been proposed to influence condensate dynamics, although its precise role remains under debate (Jayabalan *et al*., 2016; Markmiller *et al*., 2019). Under stress conditions, glycosylation of ribosomal subunits facilitates the recruitment of the translational initiation complex into SGs in human cells (Ohn *et al*., 2008). Finally, methylation of arginine residues within the RGG domains of several proteins has been found to repress SG assembly (Tsai *et al*., 2016; Hofweber *et al*., 2018; Lenard *et al*., 2021). While this modification has not yet been shown to affect condensates in plants, it is known to play important roles in plant development (Wang *et al*., 2007; Deng *et al*., 2010; Hu *et al*., 2019; Agrofoglio *et al*., 2024; Barre‐Villeneuve *et al*., 2024; Barré‐Villeneuve & Azevedo‐Favory, 2024).

## Conclusions and future directions

In the past few years, important progress has been made in the dynamics of cytoplasmic biomolecular condensates in plants, revealing a myriad of constituents present in these aggregates. While many factors were proposed to be involved in condensate formation, the PTM of these factors appears as an additional layer of regulation. In particular, several enzymes involved in PTMs were identified, such as kinases, ubiquitination components, lysine deacetylases, and redox regulators, but their contribution to biomolecular condensate dynamics is far from being understood. The consequence of these modifications on protein structure, RNA‐binding activities, and association with other partners within condensates will need further investigation. Interestingly, some examples (e.g. TZF1 and NPR1) document the contribution of different types of PTMs in regulating their localization to biomolecular condensates. How are these different mechanisms coordinated? Are they specific to distinct stress conditions? Moreover, additional PTMs (e.g. glycosylation and Arg methylation) arising in biomolecular condensates in mammals might also be discovered in plants (Wang *et al*., 2023). The recent development of enrichment methods for analyzing specific PTMs will likely enable better characterization of the role of novel PTMs in condensate dynamics (Brandi *et al*., 2022). In addition to proteins, mRNA molecules also play a role in the dynamics of condensates (Bounedjah *et al*., 2014). Like proteins, mRNAs can undergo chemical modifications, such as N6‐methyladenosine (m6A). This modification has recently been proposed to mediate RNA localization within SGs in plants and may be reversible, possibly due to the presence of demethylases localized in SGs (Fan *et al*., 2025). However, how chemical modifications of mRNAs and proteins are coordinated to regulate condensate dynamics remains an open question. Hence, a deeper characterization of the mechanism leading to cytoplasmic biomolecular condensate formation would allow better understanding of the exact functions of these distinct aggregates. Finally, our current understanding of the crosstalk between condensate dynamics and PTMs remains largely descriptive, with significant uncertainty surrounding the causal relationships between both mechanisms. To address this gap, it is crucial to better investigate the motifs (such as IDRs) necessary for phase separation and examine their potential PTM sites and clarify their regulatory roles and biological functions. Interestingly, the presence of many metabolic enzymes found in biomolecular condensate proteomes might reveal a role of their compartmentation to fine‐tune the metabolism under developmental or stress constraints. Recently, new techniques have been developed in plants, such as the Targeted Condensation‐prone‐protein Degradation system, which is based on the fusion of a targeted protein to an E3 ubiquitin ligase that induces self‐degradation after condensation (also called E3TCD1). This technique could also be a good strategy to better characterize the key players involved in condensation formation and the physiological importance of these aggregates in plant development and stress response (Luo *et al*., 2025).

## Competing interests

None declared.

## Author contributions

ML, JPR and RM wrote the manuscript.

## Disclaimer

The New Phytologist Foundation remains neutral with regard to jurisdictional claims in maps and in any institutional affiliations.
